# Supplementary material for: Development of a computer-aided design software for the quantitative evaluation of aesthetic damage
Source: PLoS One. 2019 Dec 18;14(12):e0226322. doi: 10.1371/journal.pone.0226322 (PMC6919621; doi:10.1371/journal.pone.0226322)
Supplement: S1 Code site — 1. (ZIP) [file pone.0226322.s001.zip › [EESC_jr][Projeto]Relatorio_07_21/vendors/select2/docs/_includes/options/dropdown/filtering.html]

## Can I change when search results are loaded?

### Can Select2 wait until the user has typed a search term before triggering the request?

{% highlight js linenos %}
$('select').select2({
ajax: {
delay: 250 // wait 250 milliseconds before triggering the request
}
});
{% endhighlight %}
{% include options/not-written.html %}

### Select2 is allowing long search terms, can this be prevented?

{% highlight js linenos %}
$('select').select2({
maximumInputLength: 20 // only allow terms up to 20 characters long
});
{% endhighlight %}
{% include options/not-written.html %}

### I only want the search box if there are enough results

{% highlight js linenos %}
$('select').select2({
minimumResultsForSearch: 20 // at least 20 results must be displayed
});
{% endhighlight %}
{% include options/not-written.html %}

### How can I permanently hide the search box?

{% highlight js linenos %}
$('select').select2({
minimumResultsForSearch: Infinity
});
{% endhighlight %}
{% include options/not-written.html %}
